# Survival Disparities among Alzheimer’s Patients in Hawaii: Exploring Racial/Ethnic Disparities and Machine Learning for Survival Prediction in the Hawaii Medicare Population

**DOI:** 10.1002/alz.084478

**Published:** 2025-01-09

**Authors:** Chathura Siriwardhana

**Affiliations:** ^1^ University of Hawaii at Manoa, Honolulu, HI USA

## Abstract

**Background:**

The survival outcomes following an Alzheimer’s disease (AD) diagnosis hold significant importance for health management, caregivers, patients, and their families. Hawaii is known as the most diverse ethnic population in the United States and there exist significant racial health disparities. This study investigates racial/ethnic disparities in survival among AD patients in Hawaii and develops Machine Learning models for overall survival prediction, utilizing Hawaii Medicare data.

**Method:**

We utilized nine years of Hawaii Medicare data to gather information on developing AD after age 65, and followed them for capturing the all‐cause survival or until censoring. We examined the effects of race/ethnicity coupled with socioeconomic status (SE) on the risk of mortality. SE status was accessed by the surrogate marker: Medicare/Medicaid dual eligibility. Cox regression analysis was conducted on the overall survival while accounting for age at AD onset, Gender, and multiple comorbidities. Subsequently, a Survival Random Forest (SRF) was employed to predict survival within a machine learning framework, incorporating K years of longitudinal subject health profiles, including demographics, chronic disease profiles, observed acute conditions, and hospitalization history. A penalty‐based variable selection was employed for SRF development.

**Result:**

The study included n = 9,393 AD subjects. Our analysis revealed that American Asians (AA) had a later age at AD diagnosis (p<.001), with an average age of 85.9, compared to 82.7 and 83.3 years for Whites (WH) and Native Hawaiians and Pacific Islanders (NHPI), respectively. Low SE level exhibited an increment in mortality hazard (Hazard Ratio [HR] = 1.36, p<.001). In comparison to AA with high SE (AA + high SE), increased hazards were found for AA + low SE (1.29, p<.001), WH + high SE (1.19, p<.001), WH + low SE (1.52, p<.001), and NHPI + low SE (1.39, p<.001). The SRF model with K = 2 setting demonstrated a Concordance (C) Index of 0.806 via five‐fold cross‐validation, exhibiting robust survival predictability of AD subjects. A permutation‐based study identified a list of factors influencing subject survival.

**Conclusion:**

The onset of AD development and survival are influenced by race/ethnicity and SE status. When combined with longitudinal health data, machine learning demonstrates reasonable predictability of survival.

